# Pharmacovigilance Evaluation of the Association Between DPP-4 Inhibitors and Heart Failure: Stimulated Reporting and Moderation by Drug Interactions

**DOI:** 10.1007/s13300-018-0408-2

**Published:** 2018-03-16

**Authors:** Gian Paolo Fadini, Mayur Sarangdhar, Angelo Avogaro

**Affiliations:** 1 Department of Medicine, University of Padova, 35128 Padua, Italy; 2 Division of Biomedical Informatics, Cincinnati Children’s Hospital Medical Centre, Cincinnati, OH USA

**Keywords:** Cardiovascular disease, Disproportionality, Pharmacotherapy, Type 2 diabetes

## Abstract

**Introduction:**

In the SAVOR-TIMI trial, the risk of heart failure (HF) was increased by 27% in T2D patients randomized to the dipeptidyl peptidase-4 inhibitor (DPP4i) saxagliptin. Other studies have provided inconsistent results regarding this association. Herein, we performed a pharmacovigilance analysis of the rate of HF associated with DPP4is, focusing on stimulated reporting and moderation by drug–drug interactions.

**Methods:**

We mined the FDA adverse event (AE) reporting system (FAERS) from 2004q1 to 2017q3, including a total of 9906,642 AE reports. Rates (/1000 reports) of HF within the reports for DPP4is and reports for other antidiabetic drugs were calculated for the period up to 2013q3 (date of publication of the SAVOR-TIMI trial results) and from 2013q4 to 2017q3. Analyses were refined by filtering according to therapeutic area, concomitant diseases and drugs, and competing AEs.

**Results:**

The rate of HF among the AE reports filed for DPP4is significantly increased after 2013q3, especially for saxagliptin. When compared to non-insulin non-glitazone antidiabetic drugs, the proportional reporting ratio (PRR) of HF for DPP4is was 0.62 (95% CI 0.56–0.68) up to 2013q3 and 2.12 (95% CI 1.96–2.28) from 2013q4 to 2017q3. This stimulated reporting was consistent in subanalyses based on the presence/absence of cardiac disorders and after controlling for competing AEs. The rate of HF among AE reports for DPP4is was modestly moderated by the concomitant use of metformin (− 15%) and strongly moderated by the concomitant use of SGLT2 inhibitors (− 63%), even after excluding competing AEs.

**Conclusions:**

Within the FAERS, the association between HF and DPP4is was biased by stimulated reporting, implying that the publication of the SAVOR-TIMI trial and the subsequent regulatory warnings primed clinicians to report HF events in DPP4i users as drug-related AEs. The rate of HF associated with DPP4is was moderated when they were used in combination with SGLT2 inhibitors.

**Electronic supplementary material:**

The online version of this article (10.1007/s13300-018-0408-2) contains supplementary material, which is available to authorized users.

## Introduction

Dipeptidyl peptidase-4 inhibitors (DPP4is) are drugs that are commonly used as second-line agents for the treatment of type 2 diabetes (T2D). DPP4is can also be used as first-line agents or as monotherapy in patients who cannot tolerate metformin or for whom metformin is contraindicated, mainly due to chronic kidney disease (CKD). In phase III placebo-controlled randomized controlled trials (RCT), DPP4is have presented a safety profile comparable to that of the placebo [1], and were found to reduce the risk of cardiovascular events and all-cause mortality [2]. In longer-term placebo-controlled cardiovascular outcome trials, there was no evidence that DPP4is provide protection from cardiovascular events or mortality. In the SAVOR-TIMI trial published in September 2013 [3], into which 16,492 T2D patients who had a history of or were at risk for cardiovascular events were enrolled, the risk of hospitalization for heart failure (HF) was increased by 27% in patients who were randomized to saxagliptin compared to patients who were randomized to placebo. In a subsequent re-analysis, the risk of HF associated with saxagliptin was found to be more pronounced in the first 6 months of therapy and higher in patients with elevated baseline N-terminal BNP or with CKD [4]. In a subanalysis of the EXAMINE trial, which enrolled 5380 patients with T2D and a recent acute coronary syndrome [5], the risk of hospitalization for HR in patients with no history of HF at baseline was increased in those randomized to alogliptin as compared to those randomized to placebo [6]. However, that finding was based on a very small number of events and was not confirmed in patients with a prior history of HF, who are at the highest risk for future HF episodes. These data resulted in regulatory warnings that medicines containing saxagliptin or alogliptin might increase the risk of HF [7]. In contrast, the results of the TECOS trial, which included 14,671 T2D patients, found no risk of HF associated with the use of sitagliptin [8].

Uncertainty around the risk of HF associated with DPP4is has attracted much attention due to its huge clinical implications, given the increasingly widespread use of such drugs. Several retrospective studies of registries, administrative databases, or routinely accumulated clinical data—including millions of patients—have mostly found no enhanced risk of HF in patients receiving a DPP4i in clinical practice [9–16], although earlier studies suggested a possible increased HF risk in patients receiving sitagliptin [17]. In parallel, an analysis of spontaneous adverse event (AE) reports filed by clinicians suggested a mildly increased HF risk associated with the use of DPP4is [18]. However, it is likely that, since the publication of the results of the SAVOR-TIMI and EXAMINE trials, clinicians have been primed to report HF episodes in patients taking a DPP4i—especially saxagliptin or alogliptin—as drug-related AEs, a situation known a “stimulated reporting.”

In the study reported in the present paper, we re-analyzed the disproportional association between HF and DPP4is in one of the world’s largest pharmacovigilance databases, specifically focusing on stimulated reporting and possible mitigation by drug–drug interactions. The data that emerged from our analysis complement information from RCTs and observational studies, thus providing further guidance for clinicians on this important issue.

## Methods

We analyzed AE reports filed to the US Food and Drug Administration (FDA) AE reporting system (FAERS), which collects AE reports from all over the world. FAERS files are made publicly available on a quarterly basis as raw tables that can be mined using orthogonal database search methods. From 2004q1 to 2017q3, the FAERS included 9,906,642 AE reports. Each report coded in the FAERS contains information on the event reporting date, the demographic characteristics of the patients, the type(s) and outcome(s) of the reaction(s), the drug suspected to be responsible and concomitant drugs, along with their respective indications and duration of use, as well as the source of the report.

Safety signals emerge during the analysis of a pharmacovigilance database when an AE is reported more frequently in association with a given drug than would be expected by chance (regardless of whether the drug was reported as a suspect or as a concomitant drug), i.e., more frequently than in reports that did not refer to that drug.

To mine the FAERS, we used AERSmine [19], a web-based software package that can access reports and perform systematic normalization, unification, and ontological aggregation of drugs, clinical indications, and AEs. AEs and drug indications (reasons) for use were subjected to ontological aggregation into system organ classes, high-level group terms, high-level terms, preferred terms, and low-level terms according to the MedDRA (Medical Dictionary for Regulatory Activities) system. Text strings of drug names (brands and molecules) were mapped to generic drug names and consolidated into the ATC (Anatomical Therapeutic Chemical) ontology. To remove duplicates, individual safety report (ISR) case/version identifier number matching was implemented, retaining the most recent patient report as described within the FAERS files.

Search strings for the present analysis are defined in the Electronic supplementary material (ESM). We mined all FAERS files that were publicly available for the period from 2004q1 to 2017q3 (the last access occurred on 14 Feb 2018). Separate analyses were performed for reports filed from 2004q1 to 2013q3 and from 2013q4 to 2017q3, i.e., before or after the publication of the results of the SAVOR-TIMI and EXAMINE trials. The number of reports including HF as an AE were normalized to the total number of reports for a given drug or drug class. The 95% confidence interval (CI) of the rate/1000 reports was calculated. The HF AE was defined according to AERSmine ontological categories, which was basically superimposable on HF as defined by MedDRA terms. The proportional reporting ratio (PRR) was computed as previously described [20]. We first calculated the rates of HF in reports from the two periods that listed DPP4is (as a class or individually) and then compared those rates to the HF rates in reports filed for any other non-DPP4i drugs. The analysis was refined by only retaining reports wherein at least one drug used for the treatment of diabetes (ATC A10 class) was listed. This filter by therapeutic area is typically used to restrict an analysis to reports relating to patients who are presumably diabetic. The presence of a bias resulting from the inclusion of reports relating to patients taking drugs such as metformin, glitazones, or acarbose for the treatment of prediabetes or polycystic ovary syndrome cannot be ruled out, but it is expected to be quantitatively small. The efficiency of this approach was compared to that of using a filter based on an expanded diabetes indication, as previously described [21, 22]. In a subanalysis, we calculated HF rates and PRRs associated with DPP4is separately for reports that included and those that did not include an indication for cardiac disorders.

To study how HF rates were moderated by combining the DPP4is with other drugs, we explored the effects of metformin, SGLT2 inhibitors, and beta-blockers. Non-iron vitamins were utilized as a dummy moderator, as there is no biological rationale for protective or harmful effects of such vitamins on HF. Iron was excluded as anemia, which is a risk factor for HF, can be improved by iron supplementation [23]. We calculated HF rates among AE reports that listed DPP4is with or without the selected moderator drugs, always excluding insulin and glitazones. To account for possible diluting effects of competing AEs in drug–drug interaction analyses, we excluded the following AE–drug associations: pancreatitis for DPP4is; genitourinary tract infections (GUTI) and diabetic ketoacidosis (DKA) for SGLT2 inhibitors; bradycardia and hypotension for beta-blockers.

The statistical significance level was taken to be *p* < 0.05 and the Bonferroni correction was applied to adjust for multiple comparisons.

### Compliance with Ethics Guidelines

No ethical approval was needed for this study because it was based on publicly available data and did not include any studies with human participants or animals performed by any of the authors.

## Results

### Heart Failure Rates in Adverse Event Reports for DPP4is

**Fig. 1:**
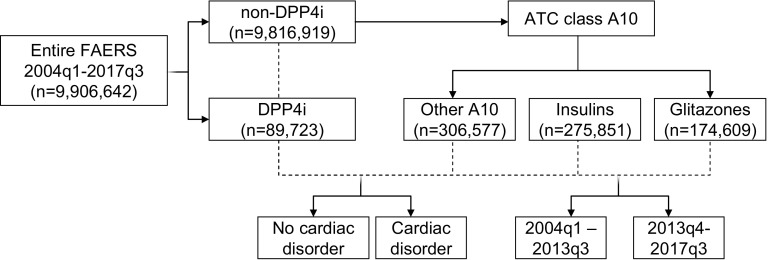
Flowchart of the study. The analytical strategy described in the “Results” section is depicted, along with the number of reports for each subgroup. Arrows indicate subgrouping, whereas dashed lines indicate comparisons

**Fig. 2:**
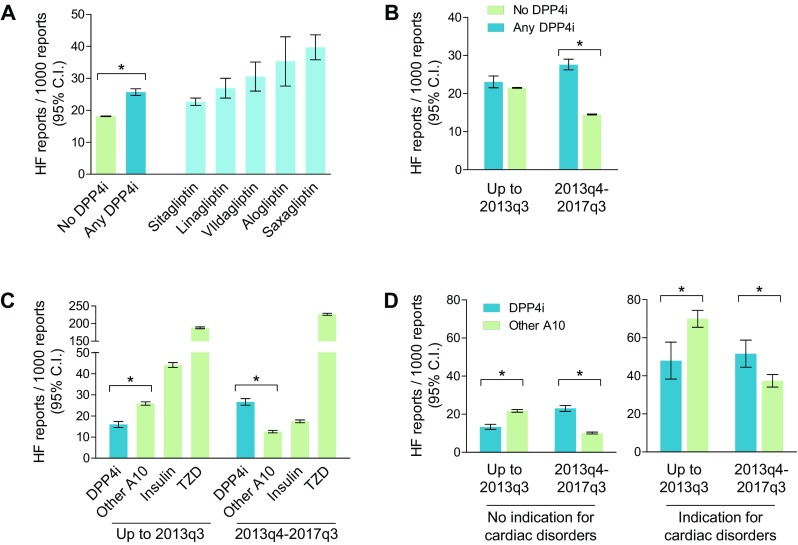
HF rates among reports listing DPP4is or other drugs. **a** HF rates in reports filed for DPP4 inhibitors (DPP4is) and in reports filed for any other drugs. The rates for the most commonly used individual DPP4is are also shown. **b** HF rates in reports filed for DPP4is and in reports filed for any other drugs during the two treatment periods (before and after 2013q3, the date of publication of the results of the SAVOR-TIMI trial). **c** HF rates among reports filed for DPP4is or for other A10 class drugs during the two periods. **d** HF rates in reports filed for DPP4is or other non-insulin non-glitazone A10 class drugs during the two periods, filtered according to the presence or absence of an indication for a cardiac disorder. In this analysis, reports listing pancreatitis as an AE were excluded. In all plots, bars indicate 95% confidence intervals (CIs). *p < 0.05 for the indicated comparison

A flowchart of the study is presented in Fig. 1. The FAERS contained 9,906,642 AE reports for the period from 2004q1 to 2017q3. There were 89,723 reports (0.9%) listing a DPP4i—used alone or in combination—as a suspect or a concomitant drug. Among these, 2303 included HF as an AE, equal to a rate of 25.7/1000 reports (95% CI 24.6–26.7). The rate was 22.7/1000 for sitagliptin, 26.9/1000 for linagliptin, 30.6/1000 for vildagliptin, 35.3/1000 for alogliptin, and 39.7/1000 for saxagliptin (Fig. 2a). Up to 2013q3 (the date of publication of the results of the SAVOR-TIMI trial), the rate of HF among reports listing DPP4is was 23.1/1000 (95% CI 21.5–24.6), but it increased significantly to 27.6/1000 (95% CI 26.2–29.0) during the period from 2013q4 to 2017q3 (Fig. 2b). As expected, the rate increased most for saxagliptin (from 21.8/1000 to 59.9/1000). This pattern was consistent with stimulated reporting.

### Heart Failure Rates in Reports Filed for Non-DPP4i Drugs

Among the 9,816,919 reports listing any non-DPP4i drug as the suspect or a concomitant drug, 177,811 included HF as an AE, equal to a rate of 18.1/1000 (95% CI 18.0–18.1). According to these crude rates, the PRR for HF associated with DPP4i was 1.42 (95% 1.36–1–48). Among reports that did not list a DPP4i, the HF rate dropped from 21.5/1000 (95% CI 21.4–21.6) during the period up to 2013q3 to 14.5/1000 (95% CI 14.4–14.6) during the period from 2013q4 to 2017q3 (Fig. 2a, b). As a result, the PRR for HF associated with a DPP4i was 1.07 (95% 1.00–1.14) for reports up to 2013q3 but 1.90 (95% CI 1.81–2.00) after 2013q3.

### Heart Failure Rates for DPP4i Versus Other Antidiabetic Medications

As diabetes is a major risk factor for HF and most of the AE reports that do not list a DPP4i relate to nondiabetic patients, the lower rate of HF in the control drug group was attributable to the dilution caused by the inclusion of reports for nondiabetic patients. Since only 54% of the reports listing an antidiabetic medication (*n* = 861,272) contained an indication of diabetes (*n* = 468,408), we filtered by therapeutic area (ATC code A10 class), assuming that reports which listed an antidiabetic medication referred to diabetic patients. Overall, the rate of HF among reports listing an A10 class drug as the suspect or a concomitant drug was 68.0/1000 (95% CI 67.4–68.5), i.e., much higher than for the reports listing a DPP4i, leading to a PRR for HF associated with DPP4is of 0.38 (95% CI 0.36–0.39). Within the A10 class, the rate of HF was markedly elevated for glitazones (206.3/1000) and moderately elevated for insulins (30.2/1000) (Fig. 2c). When the rates of HF were compared between the reports listing DPP4is and those listing other A10 class drugs (in both cases excluding reports for insulin and glitazones), the resulting PRR was 1.13 (95% CI 1.07–1.20). We used this comparison for further analyses shown below.

Since stimulated reporting was detected for DPP4i-associated HF, we quantified PRRs within the A10 class for the periods before and after 2013q3. Up to 2013q3, the rate of HF was significantly lower in reports for DPP4is than in reports for other A10 class drugs, and the PRR was 0.62 (95% CI 0.56–0.68). In the period from 2013q4 to 2017q3, the rate of HF increased among reports for DPP4is and decreased among reports for other A10 class drugs, resulting in a PRR of 2.12 (95% CI 1.96–2.28). This pattern was again consistent with stimulated reporting. Further refinement by excluding pancreatitis as a competing AE did not modify the results (not shown), but this strategy was retained in further analyses.

**Fig. 3:**
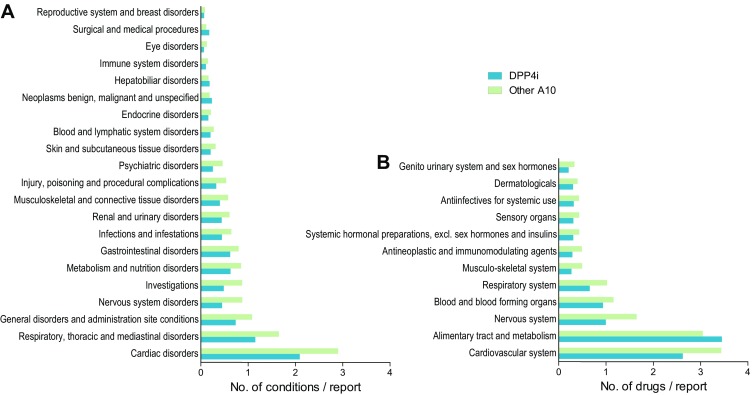
Concomitant drugs and indications. **a** Concomitant conditions—retrieved as indications for co-reported drugs—are shown for HF reports listing DPP4is or other non-insulin non-glitazone A10 class drugs, and are expressed as the number of conditions within each MedDRA category per report. **b** Concomitant drugs, grouped by system organ class, in HF reports listing DPP4is or other non-insulin non-glitazone A10 class drugs

Associated conditions were retrieved as indications for concomitant drugs in each report. The most frequent comorbidities, categorized based on MedDRA terminology, were cardiac disorders, followed by respiratory, thoracic, and mediastinal disorders (Fig. 3a).

### Heart Failure Disproportionality by Cardiac Disorder Indication

We therefore examined whether the disproportionality of HF for DPP4is differed between reports for DPP4is with an indication for a cardiac disorder versus reports for DPP4is without such an indication (Fig. 2d). In the entire FAERS, 4.0% of reports included an indication for a cardiac disorder, and the rate of HF in those reports was significantly higher (48.3/1000) than the rate in reports without an indication for a cardiac disorder (16.9/1000). Overall, 7.9% of the reports for a DPP4i and 8.2% of the reports for another A10 class drug included an indication for a cardiac disorder. Among the reports without an indication for a cardiac disorder, the rate of HF was 17.8/1000 for DPP4is and 15.7/1000 for other A10 class drugs, giving a PRR of 1.16 (95% CI 1.09–1.24). Conversely, among reports with an indication for a cardiac disorder, the rate of HF was 44.7/1000 for DPP4is and 50.8/1000 for other A10 class drugs, giving a PRR of 0.94 (95% CI 0.83–1.07).

When considering only reports filed up to 2013q3, before the publication of the results of the SAVOR-TIMI trial, the PRR for HF associated with DPP4is was 0.61 (95% CI 0.55–0.68) when a cardiac disorder indication was absent, and 0.69 (95% CI 0.55–0.85) when a cardiac disorder indication was present. The corresponding PRRs for the period from 2013q4 to 2017q3 increased to 1.38 (95% CI 1.17–1.63) and 2.28 (95% CI 2.10–2.49), respectively.

### Moderation of HF Rates by Drug–Drug Interactions

As expected, in the HF reports for DPP4is or for other A10 class drugs, the most commonly represented concomitant drugs related to the cardiovascular system or the alimentary tract and metabolism (Fig. 3b).

**Fig. 4:**
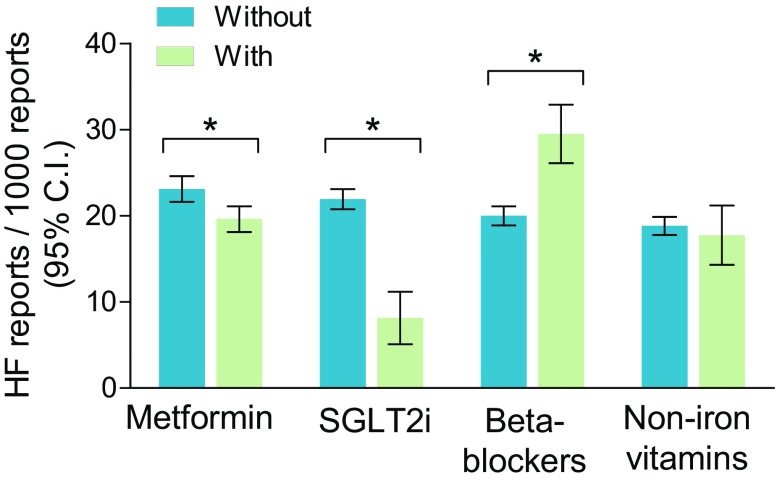
Drug–drug interactions and HF rates. The rate of HF among reports for DPP4is was calculated separately for the reports in which metformin, SGLT2 inhibitors, beta-blockers, or non-iron vitamins were co-reported and for the reports in which those drugs were not co-reported. Bars indicate 95% confidence intervals (CIs). *p < 0.05 for the indicated comparison

We examined whether the rate of HF among reports for DPP4is was modified by the concomitant presence of other medications, and focused on metformin, SGLT2i, and beta-blockers as potential moderators (Fig. 4).

Among reports for DPP4is where metformin was also listed as a suspect or a concomitant drug (47.6%), the rate of HF (19.6/1000) was slightly but significantly lower than the rate among reports for DPP4is without metformin (23.1/1000), which is equal to a reduction of 15% (95% CI 6–23%).

Among reports for DPP4is where a SGLT2i was also listed as a suspect or a concomitant drug (4.7%), the rate of HF (8.2/1000) was markedly lower than the rate among reports for DPP4is without a SGLT2i (21.9/1000), equal to a reduction of 63% (95% CI 46–75). To account for the possibility that the rate of HF in reports in which a DPP4i and a SGLT2i were listed simultaneously was due to dilution by SGLT2i-specific AEs, we refined the analysis by excluding genitourinary tract infections as competing AEs. The rate of HF remained lower in the reports for DPP4is with a SGLT2i than in the reports for DPP4is without a SGLT2i, by 61% (95% CI 43–74). Further refinement by excluding diabetic ketoacidosis as a competing AE did not change the results (not shown).

Among reports for DPP4is where a beta-blocker was also listed as a suspect or a concomitant drug (13.6%), the rate of HF (29.5/1000) was significantly higher than the rate among reports for DPP4is without beta-blockers (20.0/1000), equal to an increase of 48% (95% CI 30–68%). Excluding hypotension and bradycardia as competing AEs for beta-blockers or excluding reports with a cardiac disorder indication did not change the result.

To verify whether the presence of any co-reported drug class moderated the HF rate in reports for DPP4i, we tested non-iron vitamins, for which there is no biological rationale for a notable effect on HF. The rate of HF in reports where non-iron vitamins were co-reported with DPP4is (8.5%) was similar (17.7/1000; 95% CI 14.3–21.2) to the rate of HF among reports for DPP4is without non-iron vitamins (18.9/1000; 95% CI 17.8–19.9).

## Discussion

Several perspectives have recently highlighted that DPP4is may increase the risk of HF, although the associated mechanisms remain speculative and the evidence is uncertain [24]. In this study, we found that, among reports filed in the FAERS from 2004 to 2017, the association between HF and DPP4is was biased by strong and consistent stimulated reporting. The increased rate of HF reports for DPP4is after 2013q3 implies that publication of the results of the SAVOR-TIMI trial and the subsequent regulatory warnings primed clinicians to report HF events in DPP4i users as drug-related AEs; otherwise, given that HF is a common complication in the natural history of diabetes, HF would probably not have been perceived as a drug-related AE. Importantly, up to 2013q3, we found no evidence that HF was reported disproportionately more frequently for DPP4is than for other glucose-lowering medications, even when excluding insulin and glitazones, for which a HF signal was evident. For reports relating to patients with or without cardiac disorders, the rate of HF was significantly lower for DPP4is than for other non-insulin non-glitazone A10 class drugs up to 2013q3.

These findings contrast with a previous disproportionality analysis of the FAERS, which concluded that there was a significant HF safety signal for DPP4is as a class, as well as for the individual DPP4is [18]. From 2006q4 to 2013q4, Raschi et al. analyzed only 1,471,558 of the 4,496,095 unduplicated reports available because they filtered duplicates and excluded reports with missing data using a very conservative approach, leading them to exclude from the analysis more than two-thirds of the available data. They detected only 8963 reports for DPP4is (0.6%), while the number of available reports listing a DPP4i in the FAERS in the same period was 40,599, equal to 0.9% of the total number of reports, a rate that remained stable up to 2017q3. These technical reasons likely explain the different results obtained in the present study.

The limitations of the present study are inherent to the nature of disproportionality analyses of pharmacovigilance databases. First, the FDA does not require causal relationships between drugs and AEs to be proven (e.g., by re-challenging), so there is no certainty that the reported AE was drug-related. Second, disproportionality within the FAERS does not inform us about the true risk in clinical practice, and the PRR cannot be equated to relative risks from clinical trials or observational studies. Small disproportional AE–drug associations can achieve high statistical significance because of the huge number of records being analyzed. However, small (< 2.0) PRRs are unlikely to represent clinically meaningful signals and should be interpreted with caution and in view of the background noise. Furthermore, several potential biases have to be taken into account, such as appropriate selection of the control population, stimulated reporting, and dilution by competing AEs. Finally, several reports are incomplete and many were filed by non-healthcare professionals, including lawyers and patients themselves, which may diminish data reliability. Owing to these limitations, extreme caution should be paid when transferring the results of a pharmacovigilance assessment to the clinical setting. A guide to interpreting disproportionality analysis for glucose-lowering medications has been recently published for clinicians [25].

In addition to exploring the issue of stimulated reporting, we evaluated whether the rates of HF for DPP4is were moderated by the simultaneous presence of other medications in the same reports. An analysis of co-reported drugs did not show any clear signal that a specific drug or drug class occurred at different rates in HF reports listing DPP4is versus those not listing DPP4is. We thus based the choice of the moderator drugs on available information from the literature. In the FAERS, we detected a statistically significant but quantitatively small reduction in HF rate when metformin was co-reported with DPP4is. This finding is in line with a recent meta-analysis suggesting that cardiovascular outcomes of DPP4i-treated patients may be improved by metformin co-treatment [26], but it was too small to be considered robust. Furthermore, it should be noted that, in clinical practice, patients taking a DPP4i without metformin are more likely to suffer from CKD or respiratory disease, which are risk factors for HF and can confound the interpretation of the finding. In a re-analysis of the SAVOR-TIMI trial, the risk of hospitalization for HF in patients randomized to saxagliptin was marginally attenuated by concomitant treatment with beta-blockers [4]. In the FAERS, we found that the HF rate in reports for DPP4is in which a beta-blocker was co-reported was higher than that in reports for DPP4is that did not co-report beta-blockers, likely because beta-blocker therapy is a proxy for cardiac disease. Finally, since it is rational to combine DPP4is with SGLT-2 inhibitors, fixed-dose combinations have been developed [27], and because combinations with SGLT2is are associated with a lower risk of HF [28, 29], we examined whether HF rates were moderated in reports of a DPP4i used in combination with a SGLT2i. Interestingly, the rate of HF was reduced by > 60% by the concomitant presence of a SGLT2i, even after excluding reports of competing AEs, such as genitourinary tract infections and diabetic ketoacidosis. Since > 99% of the reports in which SGLT2is were co-reported occurred after 2013q3, this result was not affected by stimulated reporting. To control for confounding due to missing data on drugs that should have been co-reported and under-reporting, we used a dummy drug class (non-iron vitamins) that did not moderate HF risk, permitting internal validation of the usability of the system. We acknowledge that the analysis of AE moderation by concomitant drug reporting is subject to biases relating to competing AEs, under-reporting, and dilution, which can be difficult to control for. Therefore, this analysis should be considered highly exploratory.

## Conclusions

In summary, we found evidence of stimulated reporting (notoriety bias) for DPP4i-associated HF in the FAERS. However, among the reports filed before the publication of the SAVOR-TIMI trial results (threefold as many as included in a previous analysis), we found no disproportional association between DPP4is and HF. Although originating from a pharmacovigilance database, this finding could have clinical implications, as it suggests that concerns over an enhanced risk of HF due to therapy with DPP4is are unfounded. In addition, an exploratory analysis indicated that, even during the period of stimulated reporting, the rate of HF in reports for DPP4is was moderated by the presence of a SGLT2i. This favorable drug interaction needs to be tested in observational or interventional studies because confirmation of its presence would further strengthen the rationale for combining DPP4is with SGLT2is.

## Electronic supplementary material

Below is the link to the electronic supplementary material.

Supplementary material 1 (DOCX 17 kb)
